# Identification of Two Molecular Subtypes of Hepatocellular Carcinoma Based on Dysregulated Immune LncRNAs

**DOI:** 10.3389/fmolb.2021.625858

**Published:** 2021-11-23

**Authors:** Hongsheng Lin, Yangyi Xie, Yinzhi Kong, Li Yang, Mingfen Li

**Affiliations:** ^1^ Guangxi University of Chinese Medicine, Nanning, China; ^2^ Department of Clinical Laboratory, The First Affiliated Hospital of Guangxi University of Chinese Medicine, Nanning, China; ^3^ Guangxi Medical University, Nanning, China; ^4^ Department of Microbiology, School of Basic Medical Sciences, Guangxi Medical University, Nanning, China; ^5^ The First Clinical Faculty of Guangxi University of Chinese Medicine, Nanning, China

**Keywords:** long non-coding RNA (lncRNA), immune, molecular subtypes, hepatocellular carcinoma, bioinformatics

## Abstract

Long non-coding RNAs (lncRNAs) as important regulators of gene expression also have critical functions in immune regulation. This study identified lncRNA modulators of immune-related pathways as biomarkers for hepatocellular carcinoma (HCC). The profile of lncRNA regulation in immune pathways in HCC was comprehensively mapped. To determine lncRNAs with immunomodulatory functions specific to HCC, the enrichment of lncRNAs in a collection of 17 immune functions was calculated applying gene set enrichment analysis (GSEA). Unsupervised clustering of samples were performed in the R package ConsensusClusterPlus to analyze subtype survival and immunological characteristics. The enrichment of 3,134 lncRNA–immune pathway pairs in both diseased and normal samples showed a total of 1,984 immunoregulatory functional lncRNAs specific to HCC only. In addition, 18 immune-related lncRNAs were disordered in HCC and were significantly associated with immune cell infiltration. Functional enrichment analysis indicated that the 18 dysregulated immune lncRNAs were enriched in cytokines, cytokine receptors, TGFb family members, TNF family members, and TNF family member receptor pathways. Two molecular subtypes of hepatocellular carcinoma were identified based on 18 dysregulated immune lncRNAs. Immunological profiling showed that subtype 1 samples with higher levels of cytokine response had a better survival, but subtype 2 samples with higher levels of tumor proliferation had poorer survival. This study identified 18 HCC-specific dysregulated immune lncRNAs and two HCC molecular subtypes with significant prognostic differences and immune characteristics. The current findings help understand the function of lncRNAs and promote the identification of immunotherapy targets.

## Introduction

Hepatocellular carcinoma (HCC) as a highly aggressive malignancy is the third leading cause of cancer-related death (Siegel et al., 2013). Despite advances in diagnostic techniques, surgery, chemotherapy, and molecularly targeted therapy, the 5-year overall survival of HCC patients remains low due to its recurrence and metastasis (Liu et al., 2017; Innes et al., 2018). Although a number of important driver genes have been discovered for HCC management, the mechanisms through which HCC occur, metastasize, and relapse are unclear (Xiao et al., 2017; Huang et al., 2018; Sun et al., 2019). Therefore, a better understanding of the biological mechanisms underlying the onset and progression of HCC is required for the development of new diagnostic biomarkers and therapeutic strategies.

Over the past decade, the development of high-throughput RNA sequencing technologies and bioinformatics has identified about 2% of human genome sequence as protein-coding genes, and a majority of the genome are transcribed into non-coding RNAs such as small-molecule RNAs and long non-coding RNAs (lncRNAs). Early studies have primarily shown that lncRNAs are associated with a variety of cellular responses, for example, cell proliferation, apoptosis, and differentiation (Kretz et al., 2012; Wang et al., 2014). Immune system dysregulation may be a major cause of cancer development, and immunotherapy has emerged as a promising strategy for treating cancers (Kaufmann, 2019). Therefore, studying the regulation of immune gene expression is essential for generating a strong immune system. To date, most studies focused on the functions of coding genes, particularly on cell surface receptors, cytokines, and transcription factors. Recent evidence demonstrated that lncRNAs play a fundamental role in the regulation of the immune system (Chen et al., 2017; Hur et al., 2019). For example, lncRNA NeST is associated with T cell activation and has critical functions in immune response regulation. LncRNA NRON maintains T cell quiescence by isolating phosphorylated NFAT in cytoplasm (Sharma et al., 2011). Oncogenic lncRNA LINK-A down-regulates antigen presentation and intrinsic tumor suppression of cancer cells (Hu et al., 2019). However, only a few functional immune-related lncRNAs in HCC have been discovered (Jiang et al., 2017; Hu et al., 2019; Yang et al., 2019; Yu et al., 2019).

The purpose of this study was to identify immune lncRNAs specifically dysregulated in HCC and immune-related molecular subtypes of HCC, so as to provide theoretical guidance for making personalized HCC treatment. To detect immune-related lncRNAs associated with HCC, we integrated HCC data and identified lncRNAs associated with immune pathways. The immune lncRNAs screened were dysregulated in HCC and significantly associated with immune cell infiltration. Analyses of cancer-associated dysregulated immune lncRNA prioritization and cancer subtype classification demonstrated that immune dysregulation of lncRNAs was an important resource for studying their function in HCC.

## Materials and Methods

### Expression Spectral Data and Pre-Processing

**FIGURE 1:**
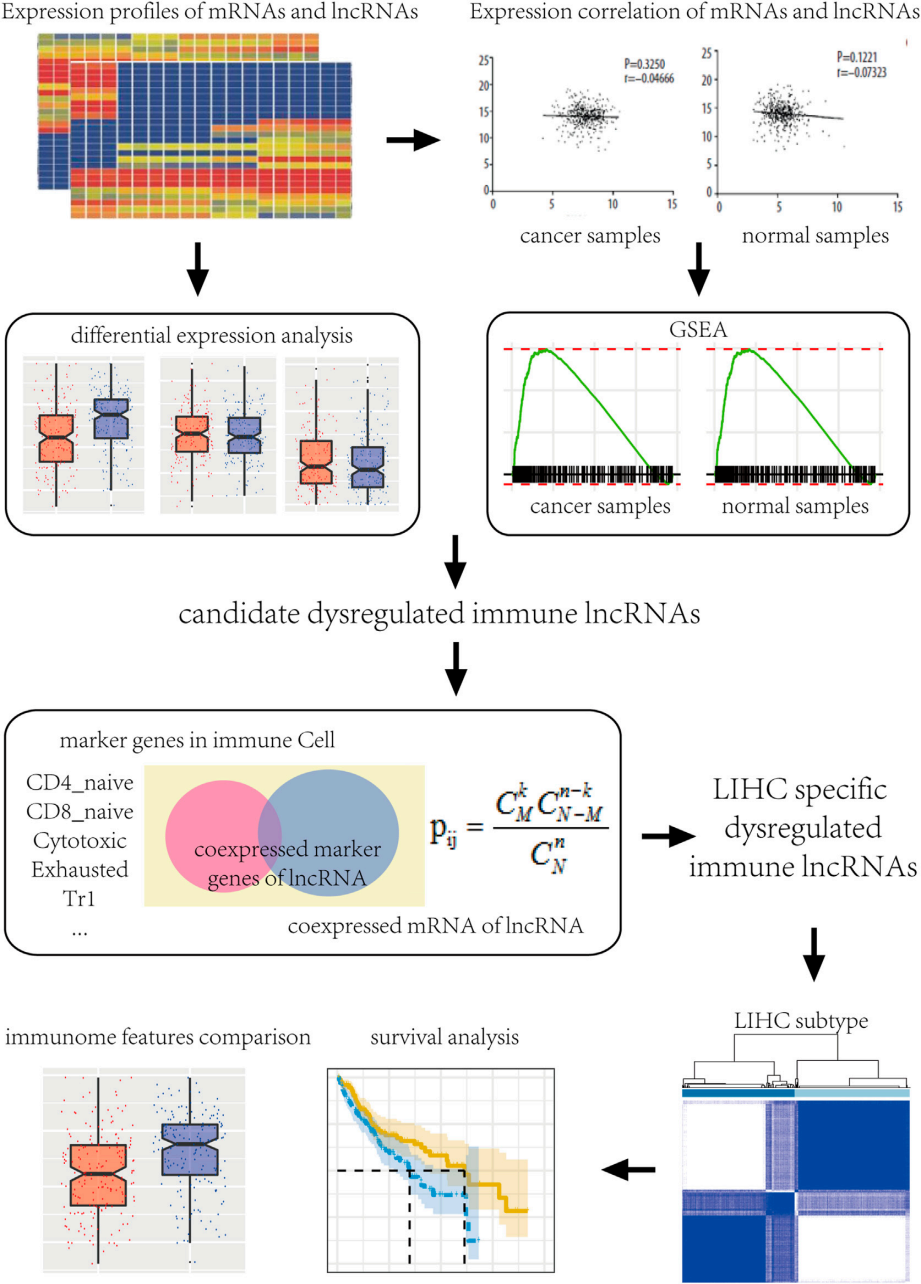
Work flow chart.

Gene expression profiles of HCC along with FPKM and count number expression profile data and clinical information of normal samples were downloaded from The Cancer Genome Atlas (TCGA) database (https://tcga-data.nci.nih.gov/) (TheLegacy. Cell (20, 2018). The expression profile was divided into lncRNA and mRNA by GENCODE gene annotation file. To retain the biologically significant genes, genes with expression values of 0 in both disease and normal samples were excluded. The work flow chart is shown in Figure 1.

### Immune Function-Related Pathways

We used the immune function collection of the ImmPort database (Bhattacharya et al., 2014), which contains a large number of human immune-related genes. We obtained a total of 17 immune function-related pathways, namely, antigen processing and presentation, antimicrobials, BCR signaling, chemokines, chemokine receptors, cytokines, cytokine receptors, interferons, interferon receptors, interleukins, interleukin receptors, natural killer cell cytotoxicity, TCR signaling pathway, TGFb family member, TGFb family members receptors, TNF family member, and TNF family member receptors. These 17 immune pathways contained a total of 1,811 related protein-coding genes available for download from Gene Lists (https://www.immport.org/shared/genelists).

### Gene Co-Expression Analysis

To investigate the relationship between gene co-expression in HCC samples and normal samples, the correlation between mRNA and lncRNA expression in the samples was calculated using Pearson’s correlation method. Logarithmic transformation of the two expression spectra was performed to ensure expression values close to normal distribution. Next, the Cor.test function in R language (Dou et al., 2020) was used to obtain the Pearce correlation coefficient and the significant *p* value of all mRNAs and lncRNAs in disease and normal samples.

### Recognition of LncRNA Regulators of Immune Function

The enrichment between lncRNA and 17 immune function-related pathways was studied using gene set enrichment analysis (GSEA) (Subramanian et al., 2007) to investigate the relationship between lncRNAs and immune function. The *R*-values and *p*-values of the correlation coefficients between the expression of each pair of mRNAs and lncRNAs in liver cancer and normal samples represented the two score values for genetic correlations with the following formula:

$$\mathrm{Score}=-\log_{10}P\times \text{sign}\left(R\right)$$

For each lncRNA, all mRNAs were ranked from small to large based on their correlation scores, and the significance of enrichment results and lncRES score (Li et al., 2020) was calculated for each pair of lncRNA and immune function-related pathway using the GSEA method. Under the condition of false discovery rate (FDR) < 0.05 and |lncRES| > 0.995, lncRNA regulators of immune function in HCC samples and normal samples were obtained.

### Differential Expression Analysis of LncRNAs

To investigate the differential expression of lncRNAs in HCC, an analysis was performed using the edgeR in R package (Robinson et al., 2010). The DGEList object was first developed through the DGEList method (DeLisi et al., 2018) with count number expression profiles of 373 disease samples and 50 normal samples. Next, genes with CPM >1 in at least two samples were retained. estimateDisp (Cheng et al., 2020) was used to estimate data dispersion, and the negative binomial generalized log-linear model (exactTest) (Shirazi et al., 2016) was used to analyze differential expression of the samples. Finally, significantly differentially expressed lncRNAs in HCC were identified with FDR < 0.05.

### Enrichment Analysis of Dysregulated Immune LncRNAs and Immune Cells

To further verify the immune role of dysregulated immune lncRNA in liver cancer, marker genes of 24 immune cells (CD4_naïve, CD8_naïve, Cytotoxic, Exhausted, Tr1, nTreg, iTreg, Th1, Th2, Th17, Tfh, Central_memory, Effector_memory, NKT, MAIT, DC, Bcell, Monocyte, Macrophage, NK, Neutrophil, Gamma_delta, CD4_T and CD8_T) were obtained from a previous study (ImmuCellAI) (Miao et al., 2020).

Next, dysregulated immune lncRNAs showing a significant correlation with marker genes of 24 immune cells were extracted, and a hypergeometric enrichment analysis method was used to identify the significant enrichment relationship between candidate dysregulated immune lncRNAs and immune cells. The hypergeometric calculation formula was as follows:

$$p_{ij}=\frac{C_{M}^{k}C_{N-M}^{n-k}}{C_{N}^{n}}$$

Specifically, *p*_ij_ represents the enrichment significance of a marker gene to an lncRNA I and immune cell J; *N* represents the number of mRNA showing significant correlation with lncRNA I in HCC samples; *M* represents the number of marker genes in immune cell J; *K* represents the number of significant correlation between lncRNA I and marker gene of immune cell J; and *N* represents the total number of mRNAs. In addition, when *K* was less than 3, the test results were not significant. Based on this method, we calculated the expression correlation and enrichment significance between each candidate lncRNA and 24 immune cells and identified a significant enrichment relationship between all dysregulated immune candidate lncRNA regulators and immune cells under the threshold of *p* < 0.05. Finally, we screened dysregulated immune lncRNAs specific to HCC and significantly enriched in at least 10 immune cells.

### Molecular Subclass Classification of HCC

According to the expression values of the dysregulated immune lncRNAs in cancer samples, the R-packs were used in unsupervised clustering of HCC samples.

### Survival Analysis of Molecular Subclasses

Survival analysis was conducted on samples according to survival time and survival status of different sample categories. Samples with survival shorter than 30 days were excluded to ensure the accuracy and validity of analysis results. Survival rate and median survival time were evaluated by the Kaplan–Meier method, and the subclasses of different samples were compared among each group based on the log-rank test.

### Analysis of Immune Characteristics of Molecular Subclasses

Differences of biological characteristics among different types of liver cancer samples were analyzed based on immunological characteristics of TCGA HCC samples (macrophage regulation, intratumor heterogeneity, proliferation, wound healing, IFN-gamma response, and TGF-beta response) from a previously published article (Thorsson et al., 2018).Wilcoxon rank sum test was used to compare biological characteristics and characterization of subclasses.

### Cell Lines and Culture

HCC cell lines HepG2, HuH7, Li7, SNU387, and SNU182 were kindly provided by the Stem Cell Bank, Chinese Academy of Sciences, and MHCC97H cells were obtained from Beyotime Company (Shanghai, China). The human liver cells THLE3 were obtained from ATCC. HepG2, HuH7, and MHCC97H cells were cultured in Dulbecco’s Modified Eagle Medium (DMEM) supplemented with 10% fetal bovine serum (FBS) and 1% penicillin/streptomycin. Li7, SNU387, and SNU182 cells were cultured in Roswell Park Memorial Institute (RPMI) 1640 medium supplemented with 10% FBS and 1% penicillin/streptomycin. Then, THLE3 cells were cultured in Endothelial Cell Medium (ECM) supplemented with 5% FBS, 1% endothelial cell growth supplement (ECGS), and 1% penicillin/streptomycin. All cells were maintained in a humidified atmosphere containing 5% CO_2_ at 37°C.

### Real-Time Quantitative Polymerase Chain Reaction

Total RNA was extracted from the cells by using RNeasy Mini Kit (Qiagen, Germany). Complementary DNA (cDNA) synthesis was performed using the PrimeScript™ RT Master Mix (Takara). The primers were synthesized by Takara (Tokyo, Japan). The β-actin gene was used as an endogenous control. Quantitative polymerase chain reaction (PCR) was performed using the TB Green® Premix Ex Taq™ II (Takara) under standard conditions according to the manufacturer’s instructions and conducted with the LightCycler 480 System (Roche, Switzerland). The data were analysed using the 2^−△△Ct^ method. The primers are listed in Supplementary Table S1.

## Results

### Recognition of Immune-Related LncRNAs in HCC

In Figure 1A a total of 374 samples were included, including 373 patients with follow-up information, so a survival analysis was performed using 373 samples. The gene expression profiles of 373 liver cancer samples with 19,406 mRNA and 13,507 lncRNA and the gene expression profiles of 50 normal samples with 18,905 mRNAs and 11,770 lncRNA were obtained.

**FIGURE 2:**
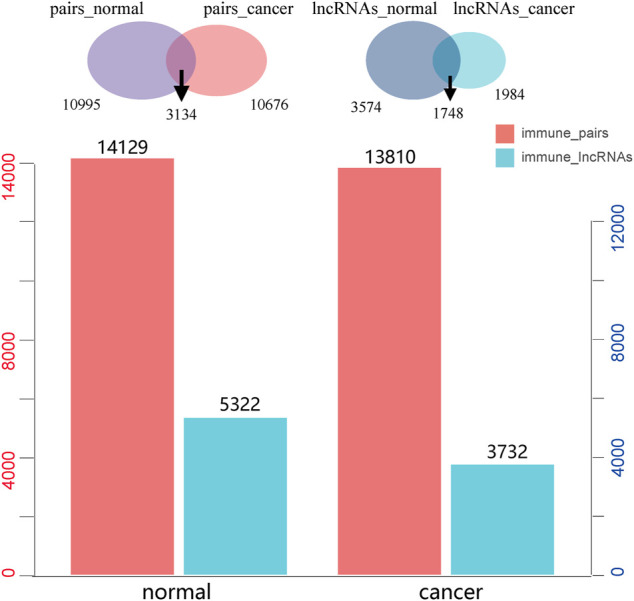
The number of long non-coding RNA (lncRNA)–immune pathway pairs recognized in liver cancer and normal samples and the number of immunological function lncRNAs, in which red represents lncRNA–immune function pairs and blue represents lncRNAs enriched in immune function.

We identified 13,810 significant lncRNA–immune function pathway pairs in HCC samples, and there were 3,732 lncRNA regulators enriched to different immune function sets. Meanwhile, 14,129 significant lncRNA–immune function pathway pairs were detected in the normal samples; here, 5,322 lncRNAs were found to be enriched to different immune functions. Furthermore, a comparison of the immune function of lncRNA regulators in normal and HCC samples showed the enrichment relationships of 3,134 lncRNA–immune pathways in both diseased and normal samples; specifically, HCC and normal samples account for 22.7% and 22.04% of the total lncRNA–immune pathway pairs, respectively (Figure 2). In addition, 1,748 lncRNAs showed immunoregulatory functions in both cancer and normal samples, accounting for 46.84% and 32.84% of all the immunoregulatory functions, respectively, and a total of 1,984 immune-regulating lncRNAs were only found in HCC (Figure 2). These results indicated that the immune function of lncRNAs was significantly changed during cancer progression; therefore, we further focused on analyzing those lncRNAs with immune functions only in HCC samples.

### Identification of Immune Pathway-Enriched LncRNAs

**FIGURE 3:**
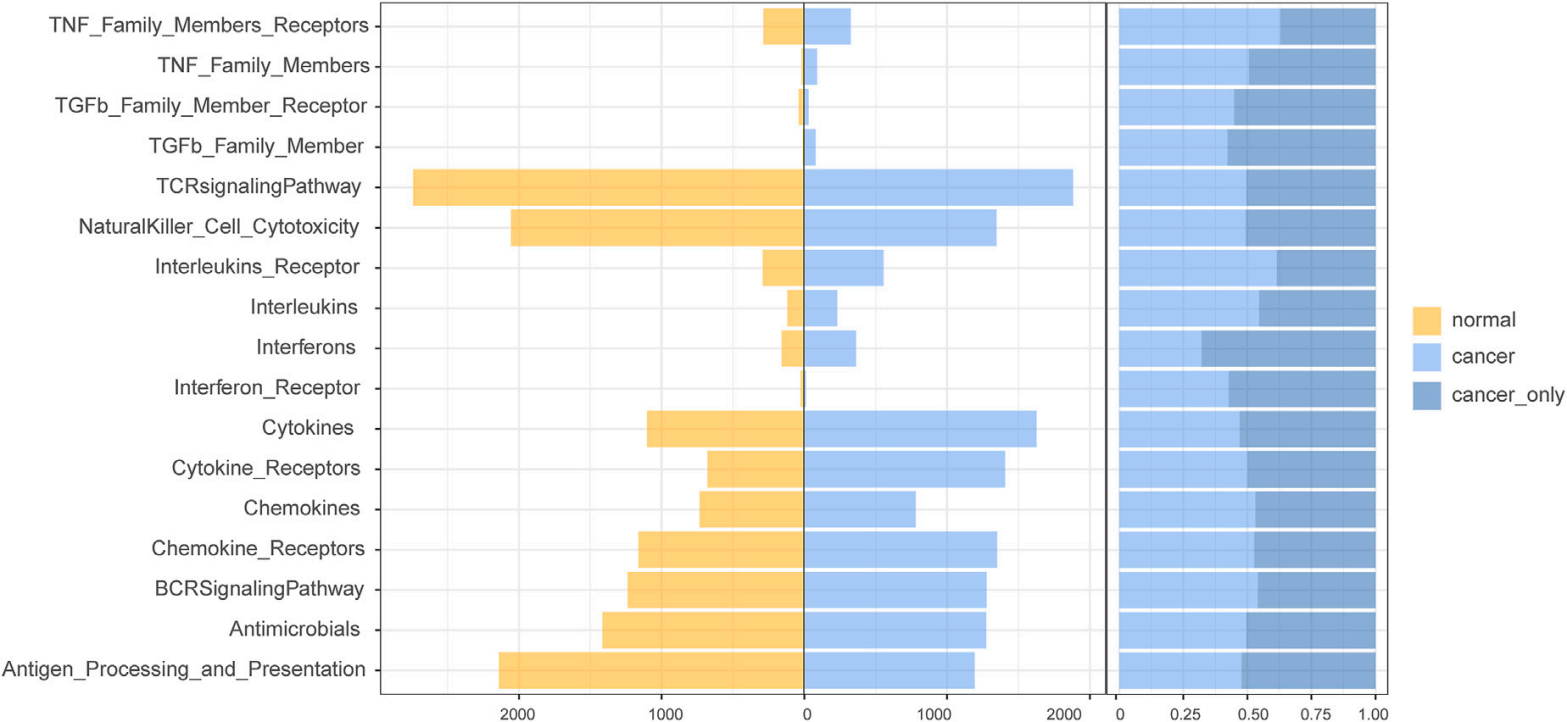
The number of immunofunctional pathway-enriched lncRNA in hepatocellular carcinoma (HCC) and normal samples.

After comparing the quantity of lncRNAs enriched in different immune pathways in normal and cancer samples, we found that more lncRNA regulators were enriched in cytokines, cytokine receptors, interleukins, and interleukins receptors in HCC samples (Figure 3). In addition, a higher proportion of HCC-specific immune function lncRNAs such as cytokines and interferons were involved in the regulation of immune-related molecular pathways (Figure 3).

### Identification of Candidate Aberrant Immune LncRNAs Specific to HCC

**FIGURE 4:**
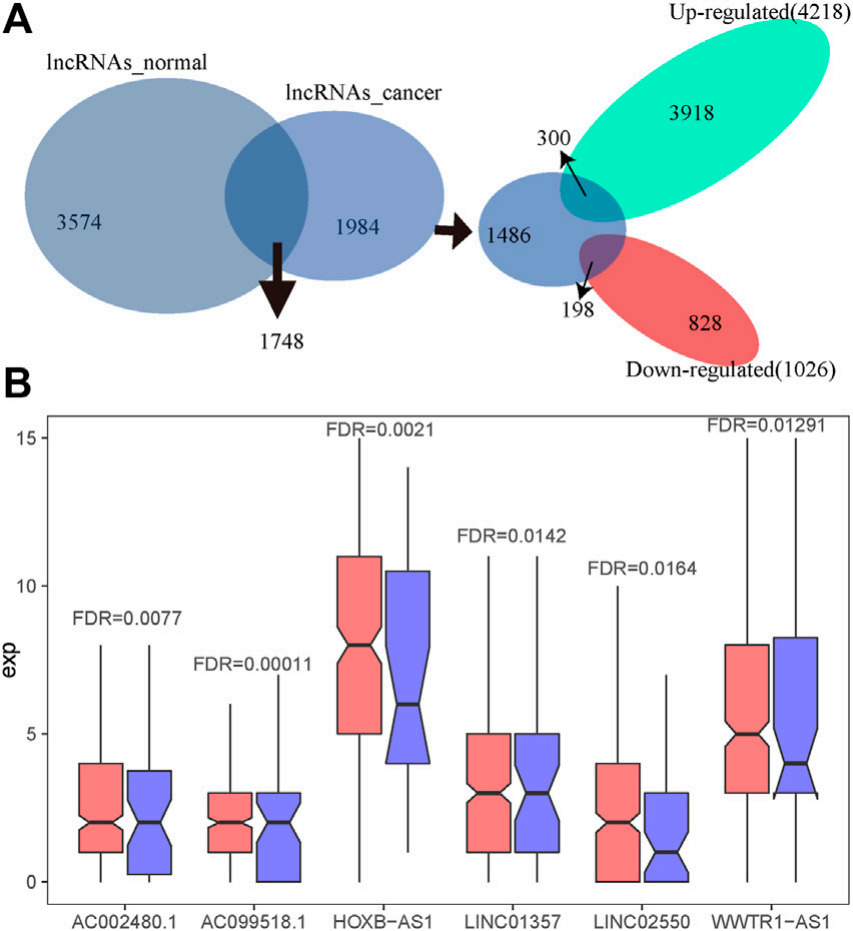
The identification of candidate aberrant immune lncRNAs specific to HCC. **(A)** Intersection of differentially expressed lncRNAs and lncRNAs of cancer-specific lncRNA–immune function pathway pairs. **(B)** The expression of lncRNAs in HCC and normal tissues, in which red represents the expression distribution of lncRNAs in cancer and blue represents the expression distribution of lncRNAs in normal samples.

As most genes with a key role in cancer development show dysregulated expression, we performed a differential expression analysis on the count expression profile of liver cancer from TCGA. Under the threshold of FDR < 0.05, we identified 5,244 significantly differentially expressed lncRNAs (4,218 highly expressed lncRNAs and 1,026 significantly lowly expressed) in HCC, accounting for 39.3% of all lncRNAs. Next, the intersection of these 5,244 significantly dysregulated lncRNAs and 1,984 lncRNAs specific to immune function of HCC showed a total of 498 lncRNA regulators with both characteristics, that is, significant expression differences in liver cancer samples and immune function specific to HCC. These 498 candidate lncRNA regulators included 300 significantly up-regulated lncRNAs and 198 significantly down-regulated lncRNAs in HCC (Figure 4A). Six of these lncRNAs were significantly dysregulated in liver cancer (Figure 4B).

### Functional Enrichment Analysis

**FIGURE 5:**
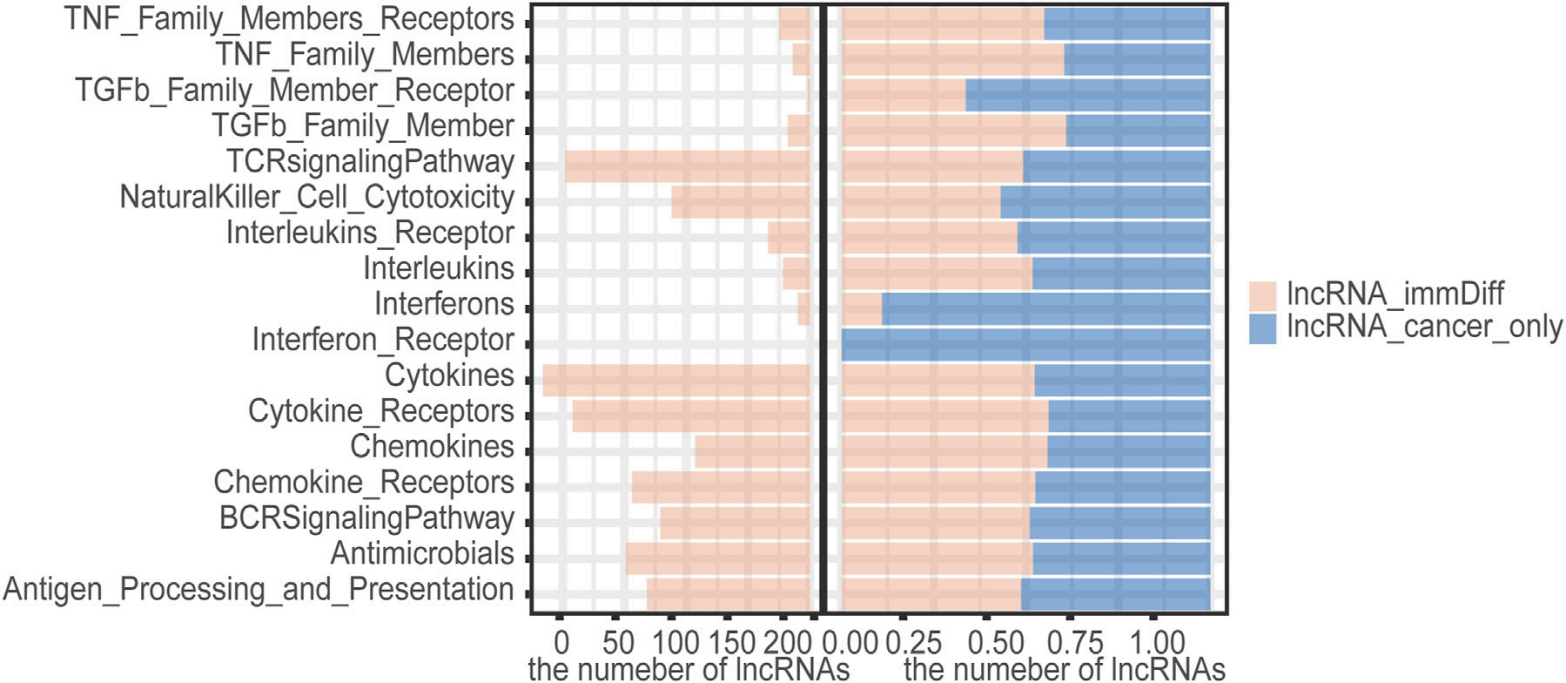
The number of candidate aberrant immune lncRNAs in the immune pathway and the ratio of candidate aberrant immune lncRNAs in the immune pathway to HCC-specific lncRNAs.

We found that 498 differentially expressed immune function lncRNAs regulated a total of 17 immune molecular pathways, involving 1,482 lncRNA–immune function pathway relationship pairs, with an average of 93 candidate lncRNAs enriched in each pathway. Among them, more lncRNAs were enriched in cytokines, cytokine receptors, and TCR signaling pathway. Compared with liver cancer-specific immune function lncRNAs, dysregulated immune lncRNAs were more involved in cytokines, cytokine receptors, TGFb family member, TNF family member, and regulation of TNF family member receptor pathway (Figure 5). Cytokines mediate the interaction between cells and have a variety of biological functions, such as regulating cell growth, differentiation, and maturation; function maintenance; regulating immune response; participating in inflammatory response and wound healing, and a wide range of anti-tumor activities. TGFb family member enhances tumor migration and inhibits immune cell function by influencing the tumor microenvironment. Therefore, these candidate immunocompromised lncRNAs may play an important role in tumor invasion and metastasis.

### The LncRNAs Were Associated With Immune Cell Infiltration

**FIGURE 6:**
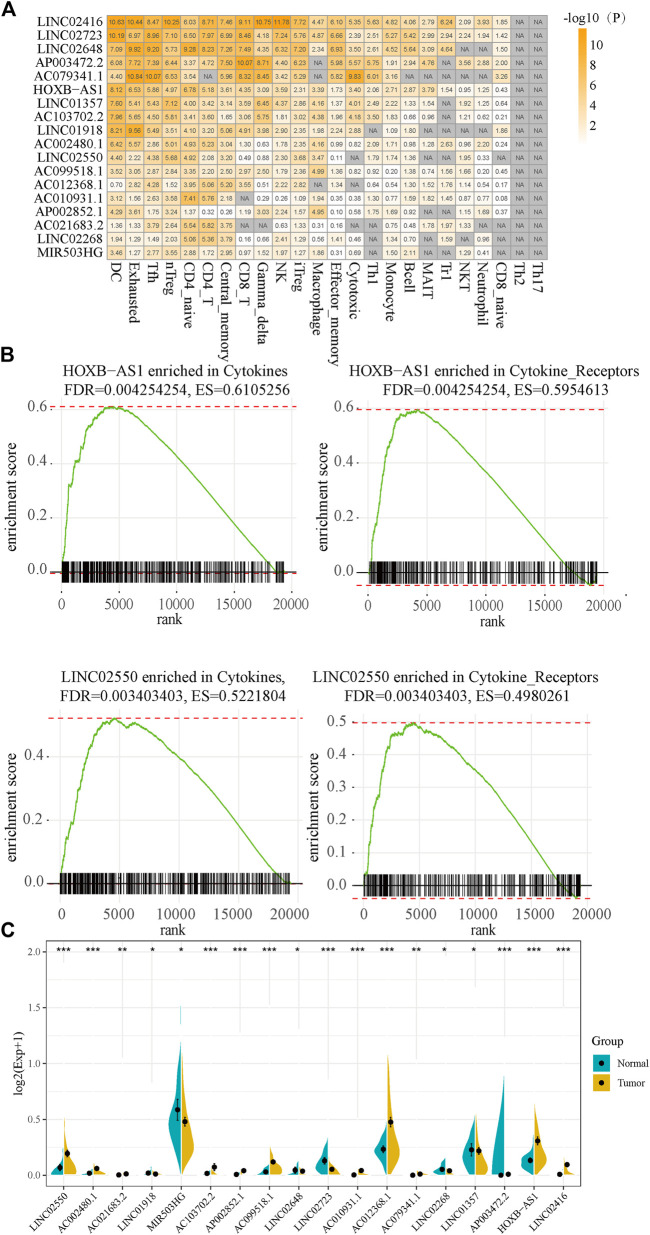
The functional analysis of dysregulated immune lncRNA. **(A)**. Enrichment significance of dysregulated immune lncRNAs and immune cells. **(B)** HOXB-AS1 and LINC02550 are enriched for cytokines and cytokine_self pathways. **(C)** The expression of 18 lncRNAs in cancer and adjacent cancer.

To further examine immune regulation of candidate lncRNAs in liver cancer, we collected marker genes of 24 immune cells. Among them, effector memory cells contained 32 marker genes, with an average of 19 marker genes in each immune cell. We obtained a total of 538 significant lncRNA–immune cell relationship pairs, of which lncRNA LINC02723 and LINC02416 showed 22 related immune cells. Furthermore, 18 lncRNAs with a significant enrichment were considered as HCC-specific deregulated immune lncRNA collections and had at least 10 immune cells; moreover, these 18 lncRNAs were significantly enriched in 24 immune cells (Figure 6A). We found that 18 dysregulated immune lncRNAs were involved in lncRNA–immune function pathways in a total of 120 HCC samples and that T cell antigen receptor (TCR) signaling pathway, B cell antigen receptor (BCR) signaling pathway, cytokines, and cytokine receptors as well as other pathways had more lncRNA regulators. LncRNA HOXb-AS1 and LINC02550 were highly expressed in HCC and were enriched in the cytokines and cytokine_sensors molecular pathways (Figure 6B). Among the 18 lncRNAs, 12 lncRNAs were highly expressed in HCC samples, and six lncRNAs were lowly expressed in HCC samples (Figure 6C).

### The Molecular Subtypes of HCC Were Classified Based on Immune LncRNAs

**FIGURE 7:**
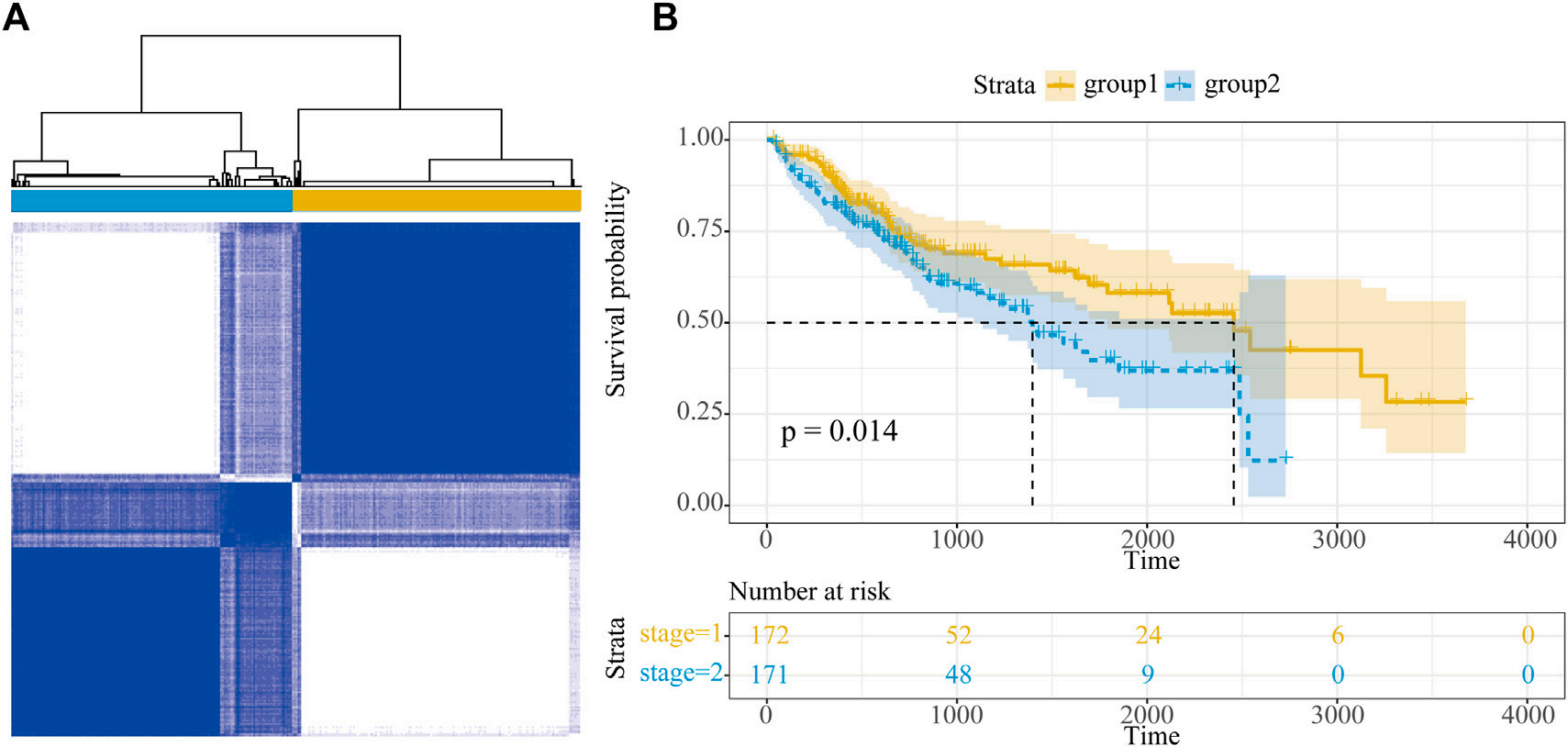
Molecular subtype of HCC. **(A)** Subtype classification of HCC samples. **(B)** Survival curve of HCC samples.

The classification of molecular subclasses of cancer samples is of great significance for personalized treatment of HCC; in this study, the samples were classified based on the expression of 18 HCC-specific dysregulated immune lncRNAs. Using the R package ConsensusClusterPlus, HCC samples in TCGA were well classified into two categories (group 1 and group 2), which contained 188 and 185 samples, respectively (Figure 7A). In addition, we integrated follow-up prognostic data, including 373 patients for prognostic analysis, and we found a significant survival difference between the two groups (*p* = 0.014), in which HCC samples in group 1 showed a higher survival rate and a longer median survival time (Figure 7B).

### Analysis of Immunological Characteristics of Molecular Subtypes

**FIGURE 8:**
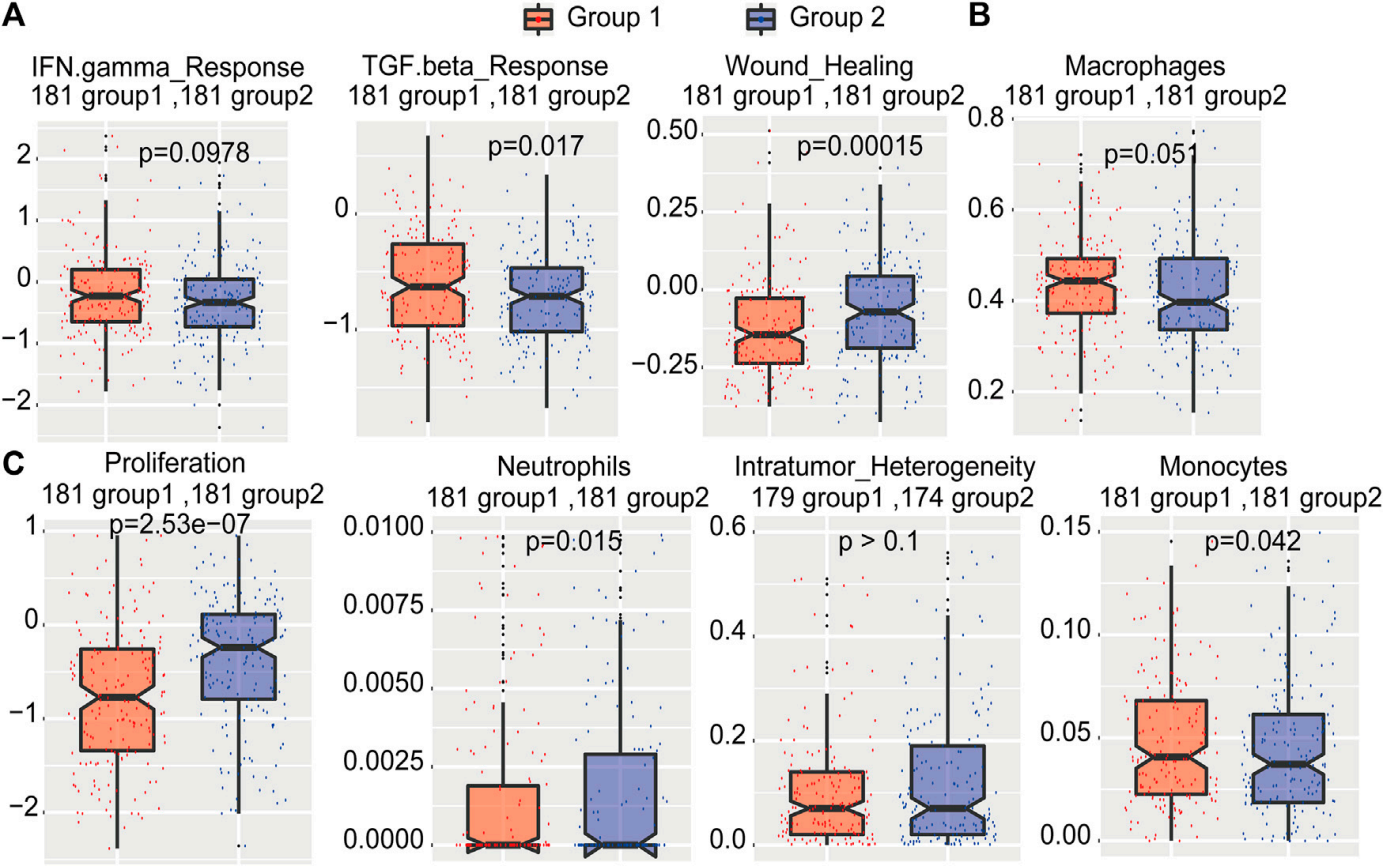
The distribution of immune characteristics of HCC subtypes.

To study the differences in immunological characteristics of these two types of samples, we obtained the scores of important immune characteristics such as intratumor heterogeneity, proliferation, wound healing, and IFN-gamma response of TCGA HCC samples. The data demonstrated that patients in group 1 had significantly higher TGF-beta response, IFN-gamma response, and wound healing scores than patients in group 2 (Figure 8A). Moreover, group 1 patients showed significantly higher macrophage and monocyte scores than group 2 patients (Figure 8B). Compared with group 1 patients, group 2 patients had significantly higher proliferation levels, neutrophil distribution, and intratumoral heterogeneity (Figure 8C). In addition, the distribution of initial B cell, leukocyte ratio, Th1, Th17, and stromal ratios were all higher in HCC patients in group 1 (Supplementary Figure S1). These results indicated that group 1 patients had a better cytokine response and a higher percentage of immune cells, which may contribute to a better prognosis. As the samples in group 2 had higher proliferation and intratumoral heterogeneity and more neutrophil-promoting cancer metastasis, they tended to develop a poor survival.

### The Expression of LncRNA Was Verified by RT-qPCR

To verify the differences of lncRNA in different subtypes, we screened the six most relevant lncRNAs from 18 immune-related lncRNAs for experimental verification. Specifically, the classification performance of each lncRNA on two molecular subtypes was calculated for 18 lncRNAs. The lncRNA with an AUC greater than 0.6 was selected, and the optimal AIC was selected by stepwise regression analysis to obtain the optimal model. Finally, the most critical lncRNAs of the six subtypes were identified (AC002480.1, MIR503HG, AC012368.1, LINC01357, HOXB-AS1, and LINC02416). A differential expression analysis showed that AC002480.1, AC012368.1, HOXB-AS1, and LINC02416 were highly expressed, while MIR503HG and LINC01357 were lowly expressed in tumors (Supplementary Figure S2A). The expressions of six lncRNAs in HCC cell lines were analyzed by RT-qPCR. As expected, AC002480.1, AC012368.1, HOXB-AS1, and LINC02416 were highly expressed, while MIR503HG and LINC01357 were lowly expressed in HCC cell lines (Supplementary Figure S2B). These results supported the conclusion of data analysis.

## Discussion

Evidences indicate that lncRNAs are highly important for immune regulation. In this study, we identified lncRNA regulators that could modulate immune-related pathways. The analysis on the relationship between differentially expressed lncRNAs and immune cells identified a total of 18 lncRNAs with significantly enriched immune pathways, and these lncRNAs were considered as dysregulated immune lncRNAs specific to HCC. It should be noted that HCC-specific dysregulated immune lncRNAs were mainly enriched in TCR signaling pathways, BCR signaling pathways, cytokines, and cytokine receptors. In addition, we demonstrated that HCC-specific dysregulated immune lncRNAs facilitated the identification of immune-related HCC molecular subtypes and the identification of two cancer subtypes with unique immunological characteristics. Subtype 1 patients with better prognosis had a stronger cytokine response and more proportion of immune cells. The subtype 2 samples showed higher levels of proliferation, intratumoral heterogeneity, and more neutrophil-promoting cancer metastasis.

Research evidence has proven the functional importance of lncRNAs in immunity. Hu et al. (2013) identified 1,524 lncRNAs from 42 T cell samples ranging from early T cell progenitors to final differentiated T helper cell subsets. Long non-coding RNA NRON negatively regulates T cell activation by interacting with nuclear factors of activated T cells. Brazão et al. (2016) found that 20% of lncRNAs in B cells are related to enhancer or promoter regions to regulate gene transcription. LncRNAs have been identified in different B cell development stages; for example, a study showed that CTC‐436k13.6, LEF1‐AS1, SMAD1‐AS1, and MYB‐AS1 are associated with preBI, preBII, and immature cells and that OIP5‐AS, MME‐AS1, and bidirectional lncRNA CRNDE are related to more advanced development stages (Petri et al., 2015). In our study, we analyzed the enrichment of lncRNAs in immune pathways, and the results showed that dysregulated immune lncRNAs were mainly involved in cytokines, cytokine pathways, TGFb family members, TNF family members, and TNF family members, suggesting that inflammatory signal regulation may also play an important role. It has been reported that lncRNAs can regulate the expression level of proinflammatory cytokine family. LncRNA Mirt2 acts as a checkpoint that prevents abnormal inflammatory activation and is a potential regulator of macrophage polarization (Du et al., 2017). LncRNA AS1 enhances transcription of the IL-1*α* gene, and disruption of AS-1IL-1α function could restrict IL-1*α* transcription and may mitigate the damaging effects of excessive IL-1*α* in infections and inflammatory diseases (Chan et al., 19502015). Lethe is a recently discovered lncRNA that binds to the NF-κB subunit p65 (RelA), preventing the transcription of IL-6, IL-8, and SOD2 (Rapicavoli et al., 2013). These data suggested an important role of immune-dysregulated lncRNAs in the progression of HCC. In our study, we identified 18 lncRNAs with significantly enriched immune pathways as HCC-specific dysregulated immune lncRNAs and were mainly enriched in TCR signaling pathways, BCR signaling pathways, cytokines, and cytokine receptor pathways.

Many of the 18 immunocompromised lncRNAs have been reported to be associated with cancer progression in previous studies. HOXB cluster antisense RNA 1 (HOXB-AS1) has been demonstrated to facilitate the proliferation of glioblastoma cells, endometrial carcinoma, and multiple myeloma cells (Chen et al., 2019; Chen et al., 2020; Liu et al., 2020). As such, HOXB-AS1 was highly expressed in human glioblastoma tissues and cell lines and was associated with survival time of patients (Chen et al., 2019). The expression patterns of WWTR1-AS1 in human cancer remain largely unexplored. Only one study reported a significant correlation between higher expression levels of WWTR1-AS1 and larger tumor size, cervical node metastasis, and poor prognosis in head and neck squamous cell carcinoma (Li et al., 2019). LncRNA miR503HG is markedly down-regulated in HCC, and a higher expression of miR503HG could noticeably inhibit HCC invasion and metastasis *in vitro* and *in vivo* (Wang et al., 2018). Our results also provided lncRNA candidates for further investigation of lncRNA function in HCC.

However, some limitations of the study should be noted. We did not identify the target of lncRNAs, which is also critical in understanding their function. Basic experimental research and more in-depth functional research will be our future direction. As the samples lacked some clinical follow-up information, particularly some diagnostic details, therefore, we did not consider factors such as the presence of other health problems of the patient in distinguishing diagnostic biomarkers.

## Conclusion

In this study, we systematically identified the lncRNA regulators that potentially regulate immune-related pathways. The findings showed that lncRNAs were active participants in immune regulation in HCC. Notably, the immune-related lncRNAs were likely to exhibit expression perturbation in immune cells and were significantly correlated with immune cell infiltration. The immune-related lncRNAs could be applied to identify cancer subtypes with distinct immunological characterization. Our data provided a valuable resource to investigate the function of lncRNAs in immune regulation.

## Data Availability

The original contributions presented in the study are included in the article/Supplementary Material; further inquiries can be directed to the corresponding author.
