# Supplementary material for: Identification of Two Molecular Subtypes of Hepatocellular Carcinoma Based on Dysregulated Immune LncRNAs
Source: Front Mol Biosci. 2021 Nov 23;8:625858. doi: 10.3389/fmolb.2021.625858 (PMC8650115; doi:10.3389/fmolb.2021.625858)
Supplement: Supplementary file 1 [file Table1.DOCX]

**Supplementary figure legend**

**Supplementary figure 1**

The distribution of initial B cell, leukocyte ratio, Th1, Th17, and stromal ratios in patients with HCC in Group 1 were all higher.

**Supplementary figure 2**

A: Differences in expression of six lncRNAs between carcinoma and paracancer. B: The relative RNA expression of AC002480.1, MIR503HG, AC012368.1, LINC01357, HOXB-AS1 and LINC02416 in HCC cells lines was determined by quantitative real-time PCR. Mean ± SD. n =3. *P＜0.05,**P＜0.01 vs THLE3 cells. NA means that the CT (Cycle Threshold) exceeds detection limit.
