# Supplementary figures and images for: Identification of Two Molecular Subtypes of Hepatocellular Carcinoma Based on Dysregulated Immune LncRNAs
Source: Front Mol Biosci. 2021 Nov 23;8:625858. doi: 10.3389/fmolb.2021.625858 (PMC8650115; doi:10.3389/fmolb.2021.625858)

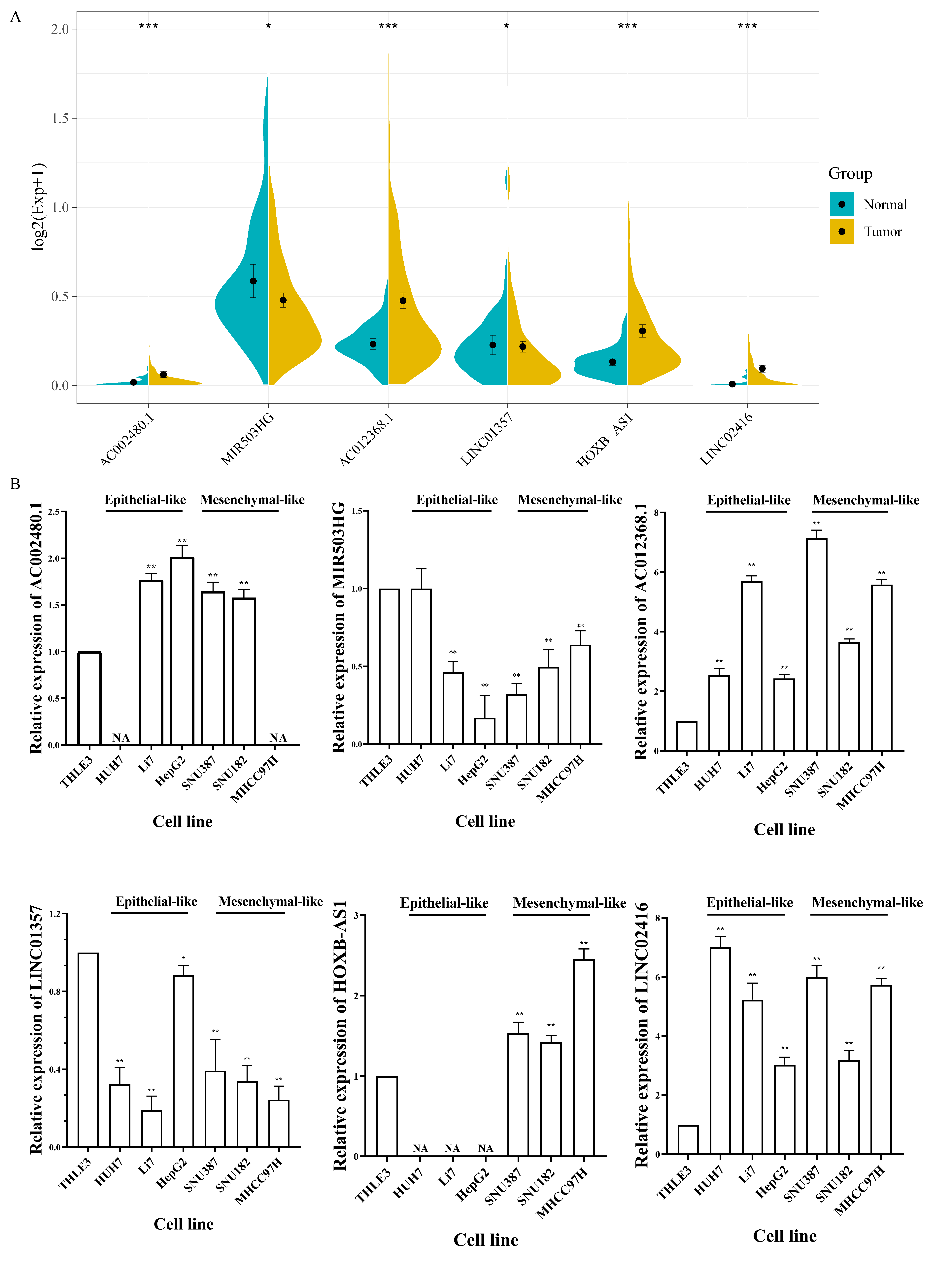

Supplement: Supplementary file 2 [file Image2.TIF]

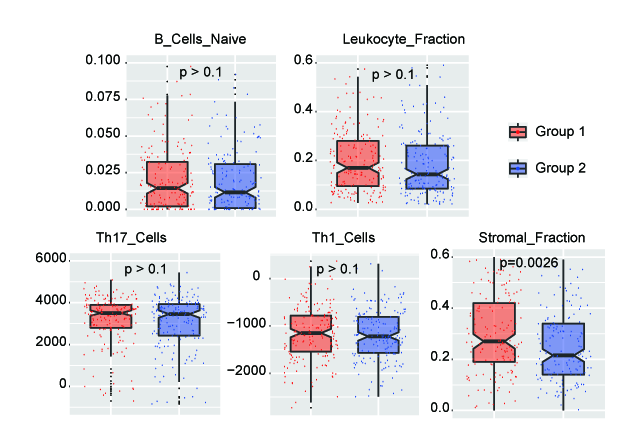

Supplement: Supplementary file 3 [file Image1.TIF]
